# Does the Contractile Capability of Pelvic Floor Muscles Improve with Knowledge Acquisition and Verbal Instructions in Healthy Women? A Systematic Review

**DOI:** 10.3390/ijerph19159308

**Published:** 2022-07-29

**Authors:** Lara Díaz-Álvarez, Laura Lorenzo-Gallego, Helena Romay-Barrero, Virginia Prieto-Gómez, María Torres-Lacomba, Beatriz Navarro-Brazález

**Affiliations:** 1 Physiotherapy in Women’s Health (FPSM) Research Group, Physiotherapy Department, Faculty of Medicine and Health Sciences, University of Alcalá, 28805 Madrid, Spain; lara.diazlv@hotmail.com (L.D.-Á.); laura.lorenzo@edu.uah.es (L.L.-G.); v.prieto@uah.es (V.P.-G.); b.navarro@uah.es (B.N.-B.); 2 Faculty of Physiotherapy and Nursing, University of Castilla-La Mancha, 45071 Toledo, Spain; helena.romay@uclm.es

**Keywords:** health education, health promotion, pelvic floor, proprioception, systematic

## Abstract

Seventy percent of women with pelvic floor dysfunctions (PFDs) are estimated to present deficient consciousness of their pelvic floor muscles (PFMs) and poor ability to contract them. Improving the proprioception of PFMs, defined as the capacity to know the status and position of each body part, and adequately contracting them could be a protective factor to prevent the appearance of PFDs in the general female population. This study aimed to identify the effectiveness of educational interventions and verbal instructions on how to contract and exercise the PFMs to improve the proprioception of the PFMs in women. A systematic search of studies published in the last 20 years until March 2022 was conducted in the PubMed, Cochrane Library, Web of Science, Scopus, PEDro, Lilacs, and Dialnet databases. A meta-analysis could not be performed due to the heterogeneity in the types of studies and included populations. This review followed the PRISMA guidelines for the design, search, and reporting of studies. The methodological quality was analysed via the PEDro and the Newcastle–Ottawa scales in the case of randomised clinical trials and non-randomised studies, respectively, while the quality of evidence was determined using the SIGN grading system for evidence-based guidelines. Descriptive and experimental studies published in English, Spanish, or Portuguese that evaluated the contractile capability of the PFMs in healthy women or women without a previous diagnosis of PFD were included. Seven articles that included a total of 2507 women were found, three of which were clinical trials with PEDro scores between 5 and 9 points out of 10 and four of which were non-randomised studies with NOS scores between 6 and 8 points out of 10. The outcomes were measured through vaginal palpation, visual observation, questionnaires for PFD symptoms, and self-perception reports. This review discriminated between two types of intervention, educational programmes and verbal instructions, and evaluated the changes observed in PFM strength and knowledgeability and the symptoms of PFDs. The findings showed that educational interventions and verbal instructions improve the proprioception of PFMs in women of all ages that are healthy or without a previous diagnosis of PFDs as well as their knowledge about the pelvic floor, healthy lifestyle habits, and symptoms that are potentially indicative of PFDs. Further high-quality randomised clinical trials are warranted to draw definitive conclusions about the effectiveness of educational interventions to improve the proprioception of the PFMs in women considered healthy or with mild symptoms that may be indicative of PFDs.

## 1. Introduction

The pelvic floor (PF) is composed of the bony, ligamentous, and muscular structures that enclose the lower region of the abdominopelvic cavity. Among its main functions are the support of the pelvic organs, the correct filling and discharge of the bladder and bowel, sphincter control, and ensuring correct sexual and reproductive function [1].

The characteristics and capacities of the PF can deteriorate throughout life because of physiological changes that take place in the female body, such as pregnancy, birth, or menopause [2]. These processes entail hormone alterations and changes in anatomy and biochemistry levels [3] that can predispose individuals to PF dysfunctions (PFD), among which the most common are urinary incontinence (UI), anal incontinence, pelvic organ prolapse (POP), and sexual dysfunctions. Over 40% of women are estimated to suffer from PFDs; the prevalence of any type of UI ranges between 25% and 45% [4], anal incontinence affects nearly 14% of parous women, even in the absence of suspected sphincter injuries [5], and 12% of women present moderate POP [6].

Despite the high prevalence of PFDs in the female population, women show poor knowledgeability of what PFDs are, their relevant risk factors, and how to manage them [7,8,9]. This lack of knowledge has been identified as one of the main barriers to women seeking and attending treatment for their PFDs and may negatively impact the development of optimal muscle characteristics of the PFMs. The PFMs are responsible for the functions of maintaining continence and allowing visceral voiding, supporting the viscera of the lesser pelvis, enabling optimal sexual and reproductive function, and contributing to lumbopelvic stability. Although PFM exercises are proposed as the strategy of first-choice treatment of both UI and mild degrees of POP, 53.2% of women are estimated to not be able to achieve the correct PFM contraction without prior training [10]. A proprioceptive deficit may be at the origin of this difficulty in achieving the isolated contraction of the PFMs by means of performing a lifting and closing movement of the urethral, vaginal, and anal orifices without activating the adjacent musculature simultaneously. Poor proprioception of the PFMs can be attributed to the lack of visual stimuli of the PF and the inability of the PFMs to deliver a large joint movement during its contraction. In addition, this deficit in sensory mechanisms combined with women’s lack of knowledge about their PF may further deteriorate proprioception over time, thereby worsening PFM function and contributing to the development of PFDs [11].

Former educational interventions in women with PFDs have usually been included as part of a physiotherapy treatment, along with PFM exercises to improve the PFM contractile capability and proprioception, alleviate relevant symptomatology, and even to prevent the further worsening of their PFDs [12,13]. Educational physiotherapy programmes include training on practical skills to correctly perform PFM exercises and learn the best muscle-strengthening strategies and how to integrate this knowledge and practical skills at home and within their daily life activities.

However, there is limited evidence of the effectiveness of this type of intervention in healthy women. The implementation of an educational intervention at early ages to provide healthy women with knowledge about the PF location and functions, its relation with PFDs, and related symptoms and signs as well as how to correctly contract and train the PFMs may help reduce the potential risks resulting from deficient proprioception and therefore prevent the development of PFDs [8].

Thus, this systematic review aimed to evaluate the effectiveness of educational interventions and verbal instructions on how to exercise the PFMs in improving the proprioception of the PFMs in healthy women or women without a previous diagnosis of PFDs who routinely attend gynaecological consultations or pelvic–perineal physiotherapy treatments.

## 2. Materials and Methods

### 2.1. Study Design

A systematic review was conducted, including randomised clinical trials (RCTs), systematic reviews, and non-randomised studies within the field of physical therapy that evaluated educational interventions about the PF region and how to exercise the PFMs in healthy women or women without a former diagnosis of PFDs who routinely attend gynaecological consultations. This review was performed in accordance with the Preferred Reporting Items for Systematic Reviews and Meta-Analyses (PRISMA) and PRISMA Protocols (PRISMA-P) statements [14]. The protocol was previously registered at the international Prospective Register of Systematic Reviews (PROSPERO) (Ref: 335033).

### 2.2. Search Strategy

Two independent researchers (L.D.A. and B.N.B.) systematically searched the PubMed, Cochrane Library, Scopus, PEDro, Lilacs, and Dialnet databases as well as the metasearch engine Web of Science between November 2021 and March 2022, entering the following keywords: pelvic floor, pelvic floor muscle, pelvic floor muscle contraction, awareness, proprioception, perception, self-perception, contraction, assessment, education, intervention, educative intervention, and educative program or educative programme. Simple searches were performed combining different terms with the AND and OR Boolean operators. The keywords were adapted to the specific characteristics of the different databases, whether in Spanish (Dialnet) or English (remaining databases). The obtained results were then filtered using humans and female (PubMed) and limited to the English, Spanish, and Portuguese languages (PubMed, Scopus, Lilacs, and Web of Science). In the PEDro database advanced search, the filters were education, health promotion, and behaviour in the therapy field. When the search produced a large number of results on Web of Science, the “Analyse results” option was utilised to limit them to the rehabilitation, health care science services, and nursing categories (Supplementary Material Table S1). A manual inverse bibliographic search of the included studies was also conducted by reviewing the studies referenced in the selected articles.

The PICO strategy was used, where the patients (P) were “healthy women”, the intervention (I) was “educational programmes”, the control (C) was either “no intervention” or “usual treatment”, although it was not always present in the selected studies, and the outcome (O) was the improvement in “proprioception/contractile capacity of the PFM”.

### 2.3. Selection Criteria

RCTs, systematic reviews, and non-randomised studies were selected, including healthy women of any age who attended a routine gynaecological consultation or physiotherapy treatment without a previous diagnosis of PFDs, evaluating the PFM contraction intra-vaginally, providing information and knowledge about the PF, and published over the previous 20 years. Studies in women with PFDs or neurological conditions and grey literature, such as PhD theses, congress summaries, protocols, case studies, and those published in languages other than English, French, Spanish, and/or Portuguese were excluded from the review. Studies were also excluded when the ratio of participants with symptoms indicative of PFDs exceeded 50% of the sample despite not having the inclusion criterion of including women with PFDs as well as when the studies lacked a descriptive analysis of the sample that hindered determining if the participants were healthy women or women with PFD symptoms. In addition, studies were excluded if the training of PFM exercises was guided intra-vaginally or with visual feedback devices. Due to the limited number of studies found, no studies were excluded on the basis of their methodological quality, which is a variable that is presented and discussed in this systematic review.

### 2.4. Selection and Data Collection Processes

Two researchers (L.D.A. and B.N.B.) independently conducted the electronic search by entering the agreed-upon terms and selected the articles based on the inclusion criteria. Several meetings were held to agree on the search results after reading the title, abstract, and methodology, and an independent evaluator (M.T.L.) intervened to reach a consensus in the case of discrepancies. Data extraction and management were performed manually without using any specific software. Finally, the methodological quality of each study was independently assessed by the two previous researchers (L.D.A. and B.N.B.), and the results were agreed upon after their individual analyses (see Table 1 and Supplementary Material Tables S2 and S3 for the consensus process).

### 2.5. Quality and Risk of Bias Assessments

The methodological quality was determined using the PEDro scale for RCTs or the Newcastle–Ottawa Scale (NOS) for non-randomised studies. The PEDro scale, with scores ranging between 0 and 10, assesses the internal validity and the statistical data of RCTs. The NOS evaluates each study based on the selection, comparability, and outcomes and assigns a value between 0 and 10 points so that the higher the score, the greater the methodological quality of the study. The AMSTAR 2 critical appraisal tool for systematic reviews was chosen to evaluate the methodological quality [22]. However, no systematic reviews were included due to them not complying with the set criteria. The Scottish Intercollegiate Guidelines Network (SIGN) scale was employed to rate the quality of evidence based on the level of quality, the risk of bias, and the type of article being analysed [23]. The JCR impact factor was also used to evaluate the influence of a journal within a specific field.

Two independent researchers (L.D.A. and B.N.B.) determined the risk of bias by evaluating the following domains in the SIGN scale: selection bias (random allocation, allocation concealment, similar baseline characteristics of participants, and participation rate); performance bias (blinding of subjects and researchers); attrition bias (high dropout rate and participants highly representative of the study population); and detection bias (follow-up of participants, the blinding of the evaluator, and the use of valid and reliable measurement instruments). The evaluators agreed upon the classification of studies as high-, low-, or very low risk, and a third assessor (M.T.L.) was consulted to resolve disagreements, if necessary.

## 3. Results

**Figure 1:**
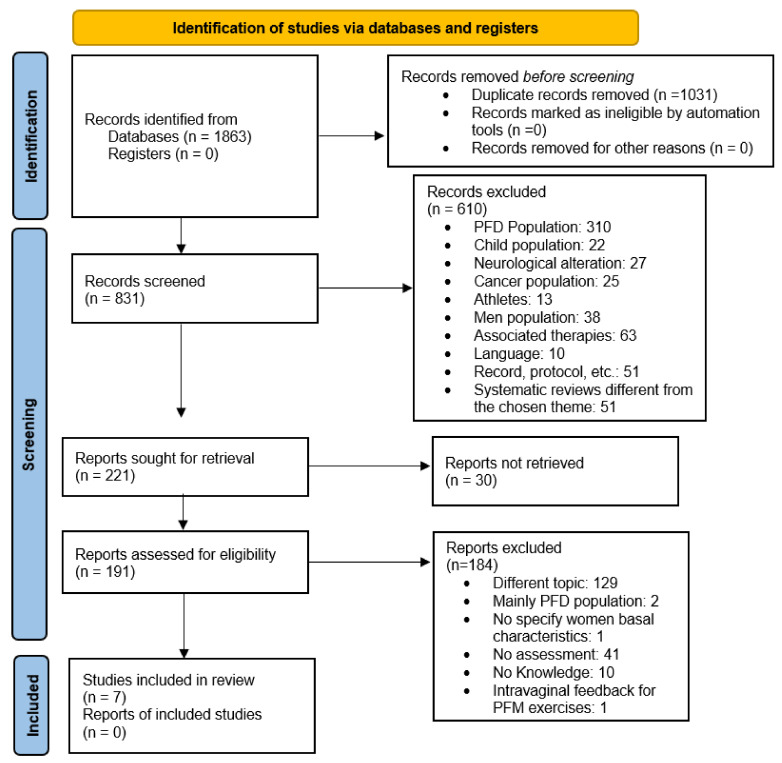
PRISMA 2020 flow diagram of the article selection procedure. PFD: pelvic floor dysfunction; PFM: Pelvic floor muscles.

The electronic search yielded a total of 1863 articles. After applying the inclusion and exclusion criteria and eliminating duplicates, seven articles were finally included: two RCTs [15,16], one non-randomised clinical trial [18], and four non-randomised studies [17,19,20,21] (Figure 1) (Table 1, Table 2 and Table 3).

### 3.1. Methodological and Scientific Quality and Risk of Bias

The quality of evidence was graded using the SIGN scale so that RCTs with a high risk of bias received a value of 1− [16,24,25] while those with high methodological quality were assigned a value of 1+ [15]. All the included non-randomised studies obtained a value of 2+ [17,19,20,21].

Overall, the studies were not accurate in detailing the randomisation and blinding methods. The randomisation tools were opaque envelopes with blocks of a maximum of 32 participants [15]. The researchers were blinded during the data analysis [15] and the assessment of outcomes [20] and blinded to the randomisation process [15] and the group allocation [15].

Only two studies carried out follow-ups with the participants every three [16] or four [15] months for a one-year period. The follow-ups were performed using self-reports by the participating women [15,16], the records of the physiotherapists who directed the educational sessions in the intervention group [15], or through voiding diaries [15,16].

All selection, performance, attrition, and detection biases were found in the included studies. The absence of randomised allocation was predominant among the detected selection biases [17,18,19,20,21]; in terms of performance bias, one RCT [16] and all the included transversal studies [17,18,19,20,21] did not blind the participants or the evaluators. In addition, the majority of studies did not blind the assessors (detection bias) [17,18,19,20,24,26]. Finally, one RCT reported a high dropout rate [16] while another did not show this information (attrition bias) [18].

### 3.2. Participants

The number of participants varied between 110 [18] and 318 [16] in the included RCTs and between 40 [17] and 958 [21] in the observational studies. The samples included women of all ages [17,19,20], during pregnancy [15,18], postpartum [20], and postmenopause [16]. No women were diagnosed with PFDs previously, although several studies detected signs indicative of the presence of PFDs that women considered to be “normal” [17], occasional UI [15,16,18,19,20,21], or POP during a baseline examination [19]. However, the number of participants with symptoms indicative of PFDs was <50% of the sample in the studies in this systematic review, and all cases were classified as mild.

### 3.3. Physiotherapy Intervention

The number of sessions in the RCTs varied between 2 [16] and 12 [15] and included pre- and post-intervention assessment, medium-term follow-ups (four weeks after the intervention) [16], and long-term follow-ups in studies in pregnant women, with measurements taken at weeks 20 and 36 during pregnancy and at 3 months [15] or 12 weeks [18] after delivery. The durations of the sessions were 10–120 min. The observational studies comprised only one session with pre- and post-intervention assessments [19,20,21] and a follow-up after 3 months [17].

The present review discriminated between two types of interventions: educational programmes [15,16,17,18] and training sessions using verbal instructions to teach how to contract the PFMs [19,20,21].

#### 3.3.1. Health Education Programmes

The educational programmes conveyed information about: PF anatomy [15,16,17,18,21] and function [16,17,18]; the types of UI and their impacts on quality of life [16]; the location of PF structures [16]; the correct contraction of the PFM [15,16,18,19,20,21] and its implementation in those activities of daily living that increase intra-abdominal pressure [18]; the importance of a healthy lifestyle [15,18], voiding habits [15] and strategies for their incorporation into daily life [15]; and the association between PFM contraction and the deep abdominal musculature [17,18].

Knowledge was transmitted verbally [17,19,20,21], with illustrations of an explanatory model [17], a video viewing of a dynamic MRI [17], or not specified [15,18]. Sampselle et al. [16] employed Bandura´s observational learning method [27], which aims to increase self-efficacy through four sources of information: verbal persuasion, emotional prompting, vicarious experiences, and performance skills.

#### 3.3.2. PFM Strengthening Programme

The PFM exercise programmes targeted young healthy women, whether pregnant, postpartum, or menopausal [15,16,18], and were performed either face-to-face [16] or in combination with home exercises [15,16]. They followed a pattern consisting of 8–12 maximal voluntary contractions of the PF held for 6–10 s, followed by 3–5 rapid contractions [15,18]. The exercises were performed in different positions [15,18], in series of two [18] repetitions of each type of contraction or twice a day [15]. Some studies combined these exercises with the concomitant contraction of the transverse abdominis muscle [17,18]; stretching of the lower limbs and lumbar spine; pelvic mobilisations; muscle strengthening exercises for the back, lower limbs, and abdomen; body awareness; breathing exercises; and muscle relaxation [15]. Women were further encouraged to perform the practised exercises during various activities of daily living that put the PF at risk, as in the case of expiratory efforts such as coughing or sneezing [17]. Sampselle et al. [16] used an audiotape that directed the training, for a minimum of three weeks, with progressions and a frequency of 50 exercises per day but did not specify what the performed exercises were.

#### 3.3.3. Verbal Instructions to Achieve PFM Contraction

Verbal instructions, which consisted of short explanations or instructions to guide the participants on how to perform a PFM contraction, were utilised in clinical trials and observational studies as an intervention method in women of all ages [19,21] and over the antenatal [15,16,18] and postpartum [20] periods.

Two studies employed verbal instructions to request participants to focus on the ability to hold urine in conjunction with squeezing and lifting the researcher’s contact at the vaginal level [19,26]. When the participating women did not understand these commands, Uechi et al. [21] indicated them to “hold urine” or “prevent the escape of gas”. The rest of the studies did not specify the verbal instruction given. In all cases, the ability to contract the PFMs was verified individually, and verbal feedback was given on how this should be performed, although the employed verbal command was not specified [15,16,17,18]. The three clinical trials included in the present review delivered the PFM training in groups [15,18] or guided by an audio tape [16], although none of them specified the verbal commands they used to ensure PFM contraction [15,16,18].

#### 3.3.4. Intervention in the Control Group

The subjects in the control groups received either no intervention [16] or were provided with routine prenatal information if the study was aimed at pregnant women [16,18].

### 3.4. Outcomes

The primary objectives of the included studies were the evaluation of the interventions’ effects on PFM strength [15,18] and PFM contraction ability [19,20,21], the self-perception of correct PFM contraction [20,21], and the perception of UI [18] and how to reduce it if it occurred over the course of the study [15,18]. The secondary outcomes were aimed at the knowledge acquired during the intervention [16] and the relationship between the self-perception of correct PFM contraction and UI-related symptoms [21].

#### 3.4.1. Contraction Ability and Strength of the PFM

The methods used to assess the ability to contract the PFM were either visual observation [16,20] or vaginal palpation, using the modified Oxford grading scale [17,18,21], the Brink scale [19], or measurement tools designed by the researcher [16]. Muscle strength was assessed by means of intravaginal manometry [15,18].

#### 3.4.2. Urinary Incontinence

Several variables related to UI were assessed, including the symptoms of urine leakage, the self-perception of improvement, the number of episodes over a period of time, and voiding frequency. Validated instruments [16,18,19,21], voiding diaries [15,16], or self-assessment [15] were used for measuring these variables.

#### 3.4.3. Knowledge about the Pelvic Floor

One study inquired about the former knowledge of the participants about the PF [20], another study excluded women who had received educational training about the PF, although it did not specify if participants were asked specific questions about their baseline knowledge [16], and two studies recorded the previous experience in performing PFM exercises as part of the baseline characteristics [15,21]. However, Sampselle et al. [16] designed a multiple-choice questionnaire to enquire about good voiding habits and the correct performance of PFM exercises that the participants in the intervention group completed post-treatment.

#### 3.4.4. Other Outcomes

Other evaluated outcomes were: the presence of POP via the POP-Quantification scale [18], the adherence to the PFM exercise programme and voiding habits through self-assessment by the participating women [16], the presence of any PFD via the Female pelvic floor questionnaire (FPFQ) for clinicians and researchers [17], and the self-perception of PFM contraction [20].

### 3.5. Effectiveness of Interventions and Adverse Effects

All educational programs significantly improved the women’s PFM contraction capability [16,18], PFM strength [15,18], and perception of UI symptoms [15,16]. An increase in knowledge about the PF was also observed [16]. The studies that included a follow-up found that women maintained their exercise regime and reported alleviated UI symptoms [15,16] and incorporated healthy lifestyle habits [16].

Observational studies found that verbal instructions yielded positive outcomes, resulting in improvements in PFM contraction capability [19]. The included studies reported that women showed deficient proprioception of PFM contraction [21], that prior knowledge or experience with PFM exercises promoted PFM contraction capability [20], and that women with POP showed greater difficulty correcting the execution of PFM contraction [19].

Only two studies [15,20] referred to possible harms of the interventions but reported an absence of adverse effects.

## 4. Discussion

Educational programmes and verbal instructions about how to contract and train the PFMs have shown their effectiveness in healthy women for improving their PFM contraction ability and strengthening the PF. Furthermore, the alleviation of mild symptomatology related to UI episodes was also reported [15,16,18,19,20,21]. The women were satisfied with the acquired knowledge and skills and deemed it important to pass them on to other women nearby [25]. The most common interventions focused on teaching concepts related to the anatomy and function of the PF, the identification of structures, the adoption of healthy habits, and guidelines for PFM training and its integration into routine moments of daily life.

Although the studies included in this systematic review addressed women without PFDs, several of them identified over the initial assessment that there were women without medical diagnoses of PFDs who also did not believe they had a problem in this body area [26]. This highlights the need to educate the general population, and women in particular, about what a PFD is and how to recognise it at an early stage in order to avoid normalising pathological processes that could receive an early treatment at low personal and economic cost and with good results [27].

Education about PF anatomy and dysfunctions, potential risk factors, and the practice of PFM exercises and their relevance in activities of daily living is unknown to most women [28]. Acquiring this knowledge and practising PFM exercises builds a concept in the brain about the contraction of this musculature. This, in turn, helps to create a movement pattern that enables women who cannot perform or find difficulties performing a PFM contraction to correctly execute it [11,28]. The socio-cultural level of the population to whom the intervention is addressed, the appraisal of the concerns and necessities of each woman [25], and therefore the individualization of the information appear to be key factors for the effectiveness and practical implementation of health education programmes [28].

The most common verbal instructions in the analysed studies referred to urine retention or squeezing the examiner’s finger. According to Kandadai et al. [29], the first indication was the most suitable for women, likely due to the high UI prevalence among them and being more familiar with it. However, the majority of participants in this systematic review did not show symptoms of UI, and most of the included studies did not specify which verbal command they employed to ensure correct PFM contraction. Some studies that were not included in this review and evaluated the PFM contraction by ultrasound obtained better results with instructions related to the rectum, such as “squeeze the anus” [30,31]. This aspect should be studied in depth since finding the best verbal indications to ensure correct PFM contraction in different populations, including healthy women, would be useful to implement educational programmes and group interventions with effective, recognisable, and easy-to-remember verbal commands that help to improve the PFM condition and thus prevent or treat PFD symptoms.

Studies that conducted a follow-up after the intervention reported women’s perseverance in terms of the newly-acquired voiding habits [16] and PFM exercises [15,16], even though the follow-up was not supervised by professionals. Periodic face-to-face follow-up meetings may contribute to promoting adherence to the exercise programme or healthy behaviours, correcting misconceptions, answering questions, or modifying exercises based on individual progression [32,33].

Several studies analysed the evolution of knowledge acquisition by the participants throughout the educational intervention [16,34] or informed about their previous knowledgeability about the PF, PFM exercise performance or experience, and instructions or feedback [15,16,20,21]. However, none assessed the initial knowledge level. Neels et al. [35,36] conducted a study to evaluate the knowledgeability of young and older women with PFD and concluded that the level was fairly low and that participants showed a need for and demanded further information about the PF. Therefore, educational interventions in healthy women are necessary to prevent PFDs, based on an assessment of their initial knowledge and providing individualised information adapted to their socio-cultural backgrounds [37], beliefs, risk factors [38], and the possibility to incorporate the acquired skills according to their health conditions [39].

The methodological and evidence quality of the studies analysed in this systematic review were rated as low to medium. Additionally, the heterogeneity in both the target populations and sample sizes was high. The main limitation was the randomisation process of the included RCTs, which was explained briefly and without detail. On the other hand, the observational studies did not inform about potential biases, sample size calculations, statistical calculations, or intermediate analyses of missing data or sensitivity, and all of them lacked a flow chart, except for the study by Uechi et al. [21].

Despite the systematic search and analyses performed, this review presents some limitations. Although most of the included studies aimed to determine the effectiveness of educational programmes and verbal instructions in healthy women, the baseline examination detected many cases of women with symptoms that could indicate the presence of mild PFDs. Therefore, studies where >50% of women presented symptoms of PFDs were excluded. Additionally, the heterogeneity in the size and characteristics of the groups of participants in the included studies and the small number of RCTs that met the inclusion criteria as well as the variety in the contents, durations, and follow-ups of the programmes and interventions among the different studies make it difficult to draw clear conclusions or to interpret or extrapolate the findings. As a result of the limited number of articles found, those with low methodological quality were not excluded. Another limitation is the manual extraction of the articles, which could have been systematised using specific software designed for this purpose. Furthermore, the evaluation of the risk of bias was conducted using the recommendations of the SIGN scale, although systematic tools are increasingly being used, such as those recommended by the Cochrane Collaboration [40].

Based on the present systematic review, implementing individualised education programmes at times of women’s vulnerability, such as during pregnancy, the postpartum period, or after menopause, could be a well-accepted preventive strategy for PFDs. They could be delivered to small groups, which could result in increased adherence to treatment and the optimisation of health resources [27,41]. In addition, the use of digital platforms could be a tool for the dissemination and easy access of knowledge for young women without PFDs [42,43], although this has not been explored in the studies included in this review. However, to draw conclusive findings, more studies are warranted to assess the effectiveness of educational interventions specifically aimed at healthy women as well as to evaluate the adherence and long-term effects and to determine the most appropriate educational method and most effective verbal commands for the development and improvement of PFM proprioception.

## 5. Conclusions

Providing information and basic notions about the PF and giving verbal instructions to guide healthy women without PFDs to perform correct PFM contractions improves their PFM contractile capability and increases their knowledge, which contributes to improving muscle performance and mild UI symptomatology. Furthermore, educational interventions help women to incorporate healthy lifestyle habits. However, given the low-to-medium level of evidence in the selected studies, further research is needed to evaluate the specific effectiveness of educational interventions about the PF in healthy women. In addition, determining their initial knowledge level about the PF would allow designing personalised programmes adapted to the specific needs of each woman.

## Figures and Tables

**Table 1:** Summary of studies included in the systematic review.

Author/s	Year	Journal	Journal Impact Factor (JCR)	City, Country	Study Design	Methodological Quality PEDro/10 and NOS /10	Evidence Grading (SIGN)
Mørkved, S. et al. [15]	2003	*Obstetrics & Gynecology*	4.965	Trondheim, Norway	Randomised clinical trial	9/10	1+
Sampselle, C.M. et al. [16]	2005	*International Urogynecology Journal*	2.094	Michigan, United States	Randomised clinical trial	5/10	1−
Talasz, H. et al. [17]	2012	*Archives of Gynecology and Obstetrics*	2.493	Innsbruck, Austria	Interventional, non-randomised, cross-sectional study	6/10	2+
Aliaga-Martínez, F. et al. [18]	2013	*Matronas Profesión*	0.123 (SJR)	Catalonia, Spain	Controlled non- randomised clinical trial	5/10	1−
Henderson, J.W. et al. [19]	2013	*Female Pelvic Medicine and Reconstructive Surgery*	1.237	Salt Lake City, Utah, United States	Observational cross-sectional study	8/10	2+
Vermandel, A. et al. [20]	2015	*International Urogynecology Journal*	2.094	Antwerp, Belgium	Observational cross-sectional study	7/10	2+
Uechi, N. et al. [21]	2019	*Neurourology and Urodynamics*	2.354	Brazil	Observational cross-sectional study	8/10	2+

JCR: Journal Citation Reports.

**Table 2:** Analysis of selected clinical trials.

Author/s	Participants	Intervention	Variables	Results
Mørkved, S. et al. [15]	N = 289 IG = 143 CG = 146 Reported incontinence during baseline examination: IG= 47 (32%) CG= 47 (31%)	Both groups were taught about PF anatomy and how to contract the PF with intra-vaginal guide. **IG**: Face-to-face treatment with a physiotherapist in groups of 10–15 women, one weekly session, 60 min, 12 weeks. MVCs of 6–8 min were requested, followed by 3–4 fast MVC. They were performed in lying, sitting, kneeling, and standing positions. At home, they must perform 8–12 MVC twice a day in the position of their choice. **CG**: customary information.	**Main variable**: Onset of UI (self-reported) **Secondary variables**: UI episodes (voiding diary), PFM contraction ability (digital palpation and observation), PFM strength (manometer).	**UI**: lower prevalence of UI in the IG that reached statistical significance at 36 weeks (32% versus 48%) and 3 months (20% versus 32%) postpartum. **Incontinence episodes**: fewer UI episodes in the IG that reached statistical significance at 36 weeks and 3 months. **PFM strength**: statistically significant difference in the IG at 36 weeks (39.9 cmH_2_O versus 34.4 cmH_2_O) and 3 months (29.5 cmH_2_O versus 25.6 cmH_2_O) postpartum. Adverse effects were not reported in the IG.
Sampselle, C.M. et al. [16]	N = 318 IG = 141 CG = 177 Reported 0–5 leak episodes during the last year: 318 (100%)	**IG**: 2 h educational group session (5–25 women) using slides and flyers about PF anatomy and physiology, UI types, and their impacts on quality of life, daily fluid intake requirement and voiding habits, how to locate and strengthen PFMs, and strategies for the incorporation of habits and exercises during DLA. Practise session of PFMT using an audio tape (3 weeks, 50 exercises each day). 10 min individual explanation to women who were not able to correctly contract PFM. **CG**: No intervention.	**Main variable**: UI (MESA questionnaire); PFM contraction ability (vaginal exam). **Secondary variables**: Knowledge (multiple-choice exam, self-produced); Voiding frequency (3-day voiding diary). Questionnaires for PFMT adherence and voiding habits at 3, 6, 9, and 12 months (self-reported).	**UI**: statistically significant difference in the IG, where 37% of women presented UI episodes versus 28% in the CG. **PFM contraction ability**: statistically significant differences in the pressure and displacement variables in the IG (68% correct contraction, 29% after instruction, and 3% failed). **Knowledge**: 90% of correct hits about voiding habits and 86% about PFMT in the IG. **Voiding frequency**: lower voiding frequency in the IG that reached statistical significance. **Adherence**: statistically significant difference in PFMT in the IG (82%, 2–3 times per week at 3 months; 68% at 12 months) and voiding habits (66% performed the recommended interval at 3 months).
Aliaga-Martínez, F. et al. [18]	N = 110 IG = 55 CG = 55 UI perception at baseline examination (ICIQ-SF = 0): IG = 42 (76.4%) CG = 39 (70.9%)	**IG**: One individual session and group sessions about anatomy, PFM function, and healthy habits at 28 and 30 weeks during pregnancy, 2 h, once a week. PFM exercises, two series per day, 8–12 contractions of 6–8 min and 3–4 fast contractions in different positions. Integration of knack manoeuvre. Eight group sessions during postpartum about protection, nursing, and PFM exercises. **CG**: customary information.	**Main variables**: PFM MVC (MOS); PFM strength and endurance (manometry); UI perception (ICIQ-SF).	**PFM MVC**: statistically significant difference in MOS. A higher percentage of women in the IG obtained a score ≥ 3 (58.2% versus 36.4%). **PFM strength and endurance**: statistically significant differences in manometry in the IG in maximum values (41.3 cmH_2_O versus 31.6 cmH_2_O), on average (28.4 cmH_2_O versus 21.5 cmH_2_O), and in contraction duration (11.6 s versus 9.4 s). **UI perception**: no statistically significant inter-group differences (92.7% without UI perception in the IG versus 81.8% in the CG).

N: number of participants; IG: intervention group; CG: control group; MVC: maximum voluntary contraction; UI: urinary incontinence; PFM: pelvic floor muscle; DLA: daily life activities; PFMT: pelvic floor muscle training; PF: pelvic floor; PFD: pelvic floor dysfunction; MOS: Modified Oxford Scale; ICIQ—SF: International Consultation on Incontinence Questionnaire—Short Form; FSFI: Female Sexual Function Index.

**Table 3:** Analysis of selected non-randomised studies.

Author/s	Participants	Intervention	Variables	Results
Talasz, H. et al. [17]	N = 40 Healthy young nulliparous women = 40 (100%) FPFQ dysfunction score (maximum 40): Mean = 10 (SD 7)	**Single group**: One group session lasting 60 min: theoretical instruction about PF anatomy and PFM function, verbal feedback with hands-on instruction about PFM exercises with co-contraction of anterolateral abdominal muscles during forced expiration and coughing. PFM exercises consisted of ten sub-maximal contractions for 10s followed by 10 fast contractions, at least 10 times per week, 3 days/week.	**Main variable**: PFM contraction ability (MOS); ability to contract PFM during coughing. **Secondary variables:** FPFQ	MOS increased post-intervention from 3.3 ± 1.7 to 4.2 ± 1.0; 72.5% performed cough-related PFM contractions; 100% reported that the acquired knowledge was helpful, and 94.6% referred the gained information to their acquaintances.
Henderson, J.W. et al. [19]	N = 779 Baseline characteristics: POP = 166 (21.3%) SUI = 133 (17.1%) POP and SUI = 35 (4.5%) No POP/SUI = 445 (57.1%)	**Only group**: Assessment of POP presence using POP-Q and PFM contraction capacity by intravaginal exam. In women performing incorrect PFM contraction, researchers repeated the assessment of the PFM contraction after additional verbal instruction.	**Main variable: PFM** contraction capacity (Brink scale) **Secondary variables**: SUI presence (Incontinence Severity Index); POP presence (POP-Q)	**PFM contraction capacity**: statistically significant difference in the group of women who presented POP and SUI, with higher proportion of women capable of contracting PFM (68.6% versus 31.4%). No statistically significant difference in groups of women with POP (86.6% versus 14.6%), with SUI (83.5% versus 16.5%), and without POP, SUI, or any of them (85.8% versus 14.2%). On a second attempt to contract PFM, 78% of 120 women who received information corrected their execution. Statistically significant difference in the group of women without POP or SUI compared to women with POP (85.7% versus 54.3%).
Vermandel, A. et al. [20]	N = 958; KEG = 500 NKEG = 458 Reported UI before pregnancy: KEG = 56 (11%) NKEG = 34 (7%) Reported UI during pregnancy: KEG = 222 (44%) NKEG = 161 (35%)	Information was collected about previous PF knowledge, its function, experience with PFM exercises, and if the participants were capable of performing a correct PFM contraction. Information was given to women that did not have former knowledge, who received personalised information and instructions on how to contract the PFMs using visual inspection.	**Main variable**: PFM contraction (visual observation) **Secondary variables**: PF knowledge (physiotherapist questions); PFM contraction awareness (physiotherapist questions)	Statistically significant difference in the performance of better PFM contraction in women with knowledge and previous experience. Comparison of women with former knowledge and experience versus others lacking knowledge or experience following physiotherapist instruction: 19.2% versus 24% improved from grade 0 to grade 1, 26.2% maintained the level versus 26.6%, and 54.6% versus 49.4% upgraded from grade 0 or 1 to grade 2, respectively. No woman worsened the performance after physiotherapist indications.
Uechi, N. et al. [21]	N = 82 Urinary incontinence self-report = 37 (45.1%)	**Only group**: First, verbal instructions about correct PFM contraction. Second, assessment of PFM contraction. Finally, analysis of women’s self-perceptions of their PFM contraction performances.	**Main variable**: PFM function (MOS) **Secondary variables**: Self-perception (correlation between participant and assessor physiotherapist MOS); UI symptoms and severity (ICIQ-UI-SF).	**PFM function**: 61% obtained a score ≥ 3 on the MOS. **Self-perception**: 33% reported correct self-perception of PFM contraction. The majority of women considered that they had a higher score compared to objective measurement. **UI symptoms and severity**: no relation was found with self-perception of PFM contraction.

N: number of participants; SD: standard deviation; FPFQ: Female pelvic floor questionnaire for clinicians and researchers; MOS: Modified Oxford Scale; PF: Pelvic Floor; POP: pelvic organ prolapse; SUI: stress urinary incontinence; PFM: pelvic floor muscle; POP-Q: Pelvic Organ Prolapse Quantification.

## Data Availability

Not applicable.

## Supplementary Materials

The following supporting information can be downloaded at: https://www.mdpi.com/article/10.3390/ijerph19159308/s1, Table S1: Search strategy by databases; Table S2: Methodological quality analysis of clinical trials measured on the PEDro scale; Table S3: Methodological quality analysis of no-randomised studies measured on the NOS scale.
