# Supplementary material for: Does the Contractile Capability of Pelvic Floor Muscles Improve with Knowledge Acquisition and Verbal Instructions in Healthy Women? A Systematic Review
Source: Int J Environ Res Public Health. 2022 Jul 29;19(15):9308. doi: 10.3390/ijerph19159308 (PMC9368630; doi:10.3390/ijerph19159308)
Supplement: Supplementary file 1 [file ijerph-19-09308-s001.zip › ijerph-1774058-supplementary.pdf]

Supplementary Material Table S1. Search strategy by databases.

DATABASE	SEARCH STRATEGY
**Common to all databases (except Dialnet)**	- Pelvic floor and assessment and educative intervention<br>- Pelvic floor and awareness and education<br>- Pelvic floor and awareness and educative intervention<br>- Pelvic floor muscle and proprioception<br>- Pelvic floor muscle contraction and awareness and education<br>- Pelvic floor muscle contraction and awareness and educative intervention<br>- (pelvic floor muscle or pelvic floor muscle contraction) and (perception or proprioception or awareness or self-perception) and female and physiotherapy
**PubMed**	- Pelvic floor and awareness and education and intervention<br>- Pelvic floor and proprioception<br>- Pelvic floor and proprioception and education<br>- Pelvic floor muscle and awareness<br>- Pelvic floor muscle and perception<br>- Pelvic floor muscle and self-perception<br>- Pelvic floor muscle contraction and assessment and education<br>- Pelvic floor muscle contraction and awareness and education and intervention<br>- Pelvic floor muscle contraction and awareness and educative program<br>- Pelvic floor muscle contraction and proprioception and education<br>- Pelvic floor muscle contraction and proprioception and education and intervention

DATABASE	SEARCH STRATEGY
**Cochrane Library**	- Pelvic floor and awareness and education and intervention<br>- Pelvic floor and proprioception and education<br>- Pelvic floor muscle and awareness<br>- Pelvic floor muscle and perception<br>- Pelvic floor muscle contraction and assessment and education<br>- Pelvic floor muscle contraction and assessment and physiotherapy<br>- Pelvic floor muscle contraction and awareness and education and intervention<br>- Pelvic floor muscle contraction and awareness and educative program<br>- Pelvic floor muscle contraction and proprioception<br>- Pelvic floor muscle contraction and proprioception and education<br>- Pelvic floor muscle contraction and proprioception and education and intervention

DATABASE	SEARCH STRATEGY
**PEDro**	- Pelvic floor and proprioception<br>- Pelvic floor muscle and awareness<br>- Pelvic floor muscle and contraction<br>- Pelvic floor muscle and contraction and awareness<br>- Pelvic floor muscle and motor control<br>- Pelvic floor muscle contraction and awareness and educative program<br>- Pelvic floor muscle contraction and awareness and educative programme<br>- Pelvic floor muscle contraction and education<br>- Pelvic floor and awareness + therapy (behaviour modification) (advanced search)<br>- Pelvic floor and awareness + therapy (education) (advanced search)<br>- Pelvic floor and awareness + therapy (health promotion) (advanced search)<br>- Pelvic floor and awareness and intervention + therapy (education) (advanced search)<br>- Pelvic floor muscle contraction and assessment + therapy (education) (advanced search)<br>- Pelvic floor muscle contraction and awareness and intervention + therapy (education) (advanced search)<br>- Pelvic floor muscle contraction and proprioception + therapy (education) (advanced search)<br>- Pelvic floor muscle contraction and proprioception and intervention + therapy (education) (advanced search)

DATABASE	SEARCH STRATEGY
**Scopus**	- Pelvic floor and awareness and education and intervention<br>- Pelvic floor and proprioception<br>- Pelvic floor muscle and awareness<br>- Pelvic floor muscle and contraction and proprioception<br>- Pelvic floor muscle and perception<br>- Pelvic floor muscle and perception and physiotherapy<br>- Pelvic floor muscle contraction and assessment and education<br>- Pelvic floor muscle contraction and awareness and education and intervention<br>- Pelvic floor muscle contraction and awareness and educative program<br>- Pelvic floor muscle contraction and awareness and educative programme<br>- Pelvic floor muscle contraction and perception<br>- Pelvic floor muscle contraction and perception and physiotherapy<br>- Pelvic floor muscle contraction and proprioception and education<br>- Pelvic floor muscle contraction and proprioception and education and intervention

DATABASE	SEARCH STRATEGY
**Web of Science**	- Pelvic floor and awareness and education and intervention<br>- Pelvic floor muscle and awareness + rehabilitation<br>- Pelvic floor muscle and perception and physiotherapy<br>- Pelvic floor muscle contraction and assessment<br>- Pelvic floor muscle contraction and assessment and education<br>- Pelvic floor muscle contraction and assessment and physiotherapy<br>- Pelvic floor muscle contraction and awareness and education and intervention<br>- Pelvic floor muscle contraction and awareness and educative program or educative programme + analyse results + rehabilitation + health care science service<br>- Pelvic floor muscle contraction and awareness and educative program or educative programme + analyse results + rehabilitation + health care science service + nursing<br>- Pelvic floor muscle contraction and proprioception and education<br>- Pelvic floor muscle contraction and proprioception and education and intervention<br>- Pelvic floor muscle contraction and (awareness or proprioception or perception) and female and physiotherapy

DATABASE	SEARCH STRATEGY
Dialnet	- Contracción and suelo pélvico and concienciación/conciencia and educación<br>- Contracción and suelo pélvico and concienciación/conciencia and educación and intervención<br>- Contracción and suelo pélvico and concienciación/conciencia and programa educativo<br>- Contracción and suelo pélvico and evaluación/valoración and educación<br>- Contracción and suelo pélvico and propiocepción and educación<br>- Contracción and suelo pélvico and propiocepción and educación and intervención<br>- Contracción musculatura suelo pélvico and concienciación/conciencia and educación<br>- Contracción musculatura suelo pélvico and concienciación/conciencia and intervención educativa<br>- Suelo pélvico and concienciación/conciencia and educación<br>- Suelo pélvico and concienciación/conciencia and educación and intervención<br>- Suelo pélvico and concienciación/conciencia and intervención educativa<br>- Suelo pélvico and evaluación and intervención educativa<br>- Suelo pélvico and musculatura and concienciación<br>- Suelo pélvico and musculatura and contracción and concienciación<br>- Suelo pélvico and musculatura and contracción and propiocepción<br>- Suelo pélvico and musculatura and control motor<br>- Suelo pélvico and musculatura and percepción<br>- Suelo pélvico and musculatura and propiocepción<br>- Suelo pélvico and valoración and intervención educativa<br>- (musculatura suelo pélvico or contracción musculatura suelo pélvico) and (percepción or propiocepción or autopercepción) and mujer and fisioterapia

DATABASE	SEARCH STRATEGY
**Lilacs**	- Pelvic floor and awareness and education and intervention<br>- Pelvic floor and perception<br>- Pelvic floor and proprioception<br>- Pelvic floor muscle and awareness<br>- Pelvic floor muscle and contraction and perception<br>- Pelvic floor muscle and perception<br>- Pelvic floor muscle contraction and assessment and education<br>- Pelvic floor muscle contraction and awareness<br>- Pelvic floor muscle contraction and awareness and education and intervention<br>- Pelvic floor muscle contraction and awareness and educative program<br>- Pelvic floor muscle contraction and awareness and educative programme<br>- Pelvic floor muscle contraction and perception<br>- Pelvic floor muscle contraction and proprioception and education<br>- Pelvic floor muscle contraction and proprioception and education and intervention

Supplementary Material Table S2. Methodological quality analysis of clinical trials measured on the PEDro scale

**PEDro items**	**1**	**2**	**3**	**4**	**5**	**6**	**7**	**8**	**9**	**10**	**11**	**TOTAL SCORE /10**
**Mørkved, S. *et al.*[17]**	Yes	Yes	Yes	Yes	Yes	No	Yes	Yes	Yes	Yes	Yes	9/10
**Sampselle, C.M. *et al.*[18]**	Yes	Yes	No	Yes	No	No	No	Yes	Yes	Yes	No	5/10
**Aliaga-Martínez, F. *et al.*[20]**	Yes	No	No	Yes	No	No	No	Yes	Yes	Yes	Yes	5/10

Supplementary Material Table S3. Methodological quality analysis of no-randomised studies measured on the NOS scale.

		Talasz, H. *et al.*[19]	Henderson, J.W. *et al.*[21]	Vermandel, A. *et al.*[22]	Uechi, N. *et al.*[23]
**Selection**	Representativeness of the sample	*	*	*	*
	Sample Size	No	No	No	*
	Ascertainment of the exposure (disease)	*	**	**	**
	Non-respondents	No	No	No	No
**Comparability**	The subjects in different outcome groups are comparable, based on the study design or analysis. Confounding factors are controlled for.	*	*	*	*
**Outcome**	Assessment of the outcome	**	**	**	**
	Statistical test	*	*	*	*
**TOTAL SCORE: /10**		**6**	**8**	**7**	**8**

or **.: identification of high quality choices. Some sections have a maximum score of one * and others of **
